# Supplementary material for: Root-specific theanine metabolism and regulation at the single-cell level in tea plants (Camellia sinensis)
Source: eLife. 2024 Oct 14;13:RP95891. doi: 10.7554/eLife.95891 (PMC11473105; doi:10.7554/eLife.95891)
Supplement: Figure 7—source data 1. [file elife-95891-fig7-data1.pdf]

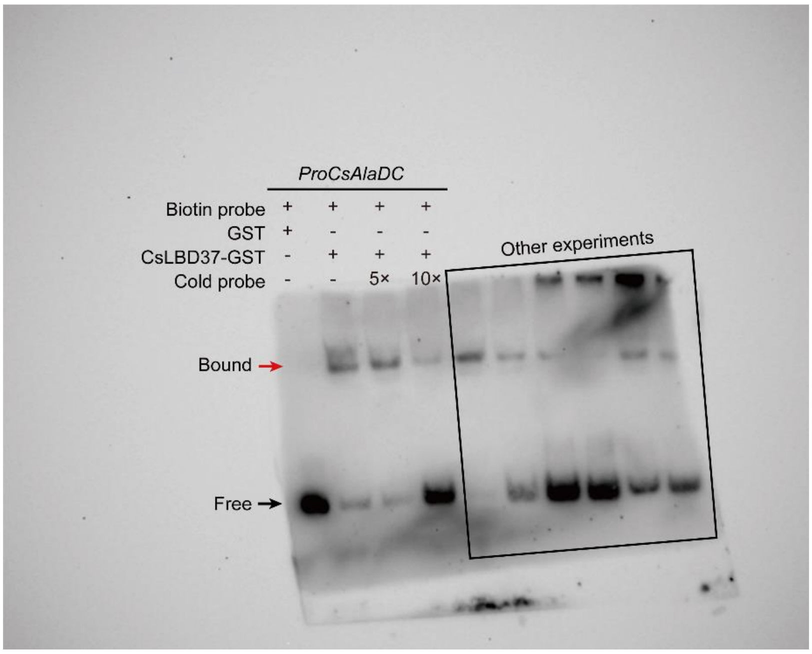

Figure 7, Source Data 1. Original membranes corresponding to Figure 7. In the first lane as control, GST protein was incubated with biotin-labeled probes. In the second lane, CsLBD37-GST protein was incubated with biotin-labeled probes. The third and fourth lane, CsLBD37-GST protein was incubated with biotin-labeled probes, and this reaction system included cold probe (without biotin labeling) at five or ten times the concentration of labeled probes. The red arrow points to the binding position, the black arrow points to the free biotin-labeled probes. The results shown in the black box belong to other experiments and are not related to this study.
