# Supplementary figures and images for: Root-specific theanine metabolism and regulation at the single-cell level in tea plants (Camellia sinensis)
Source: eLife. 2024 Oct 14;13:RP95891. doi: 10.7554/eLife.95891 (PMC11473105; doi:10.7554/eLife.95891)

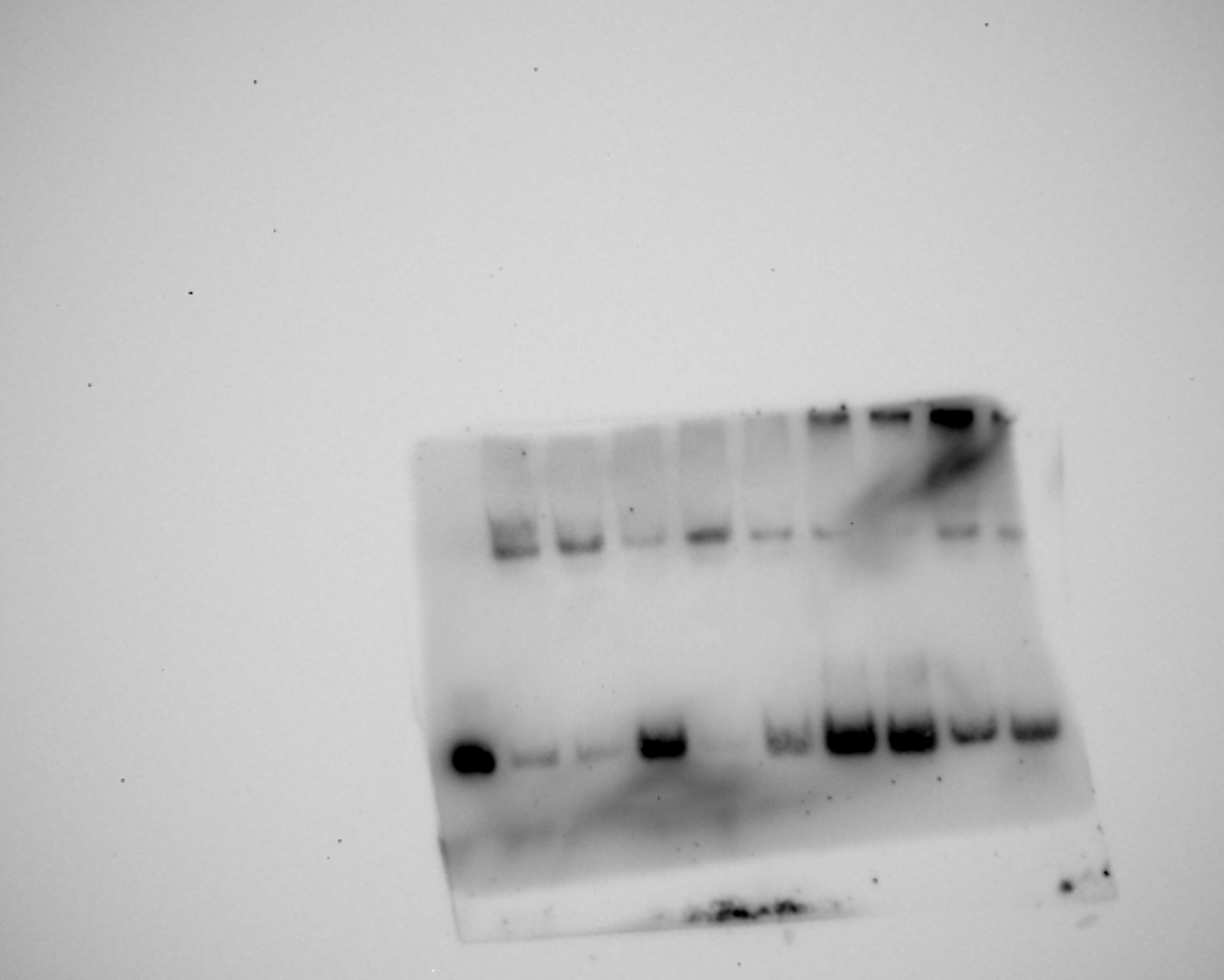

Supplement: Figure 7—source data 2. [file elife-95891-fig7-data2.tif]

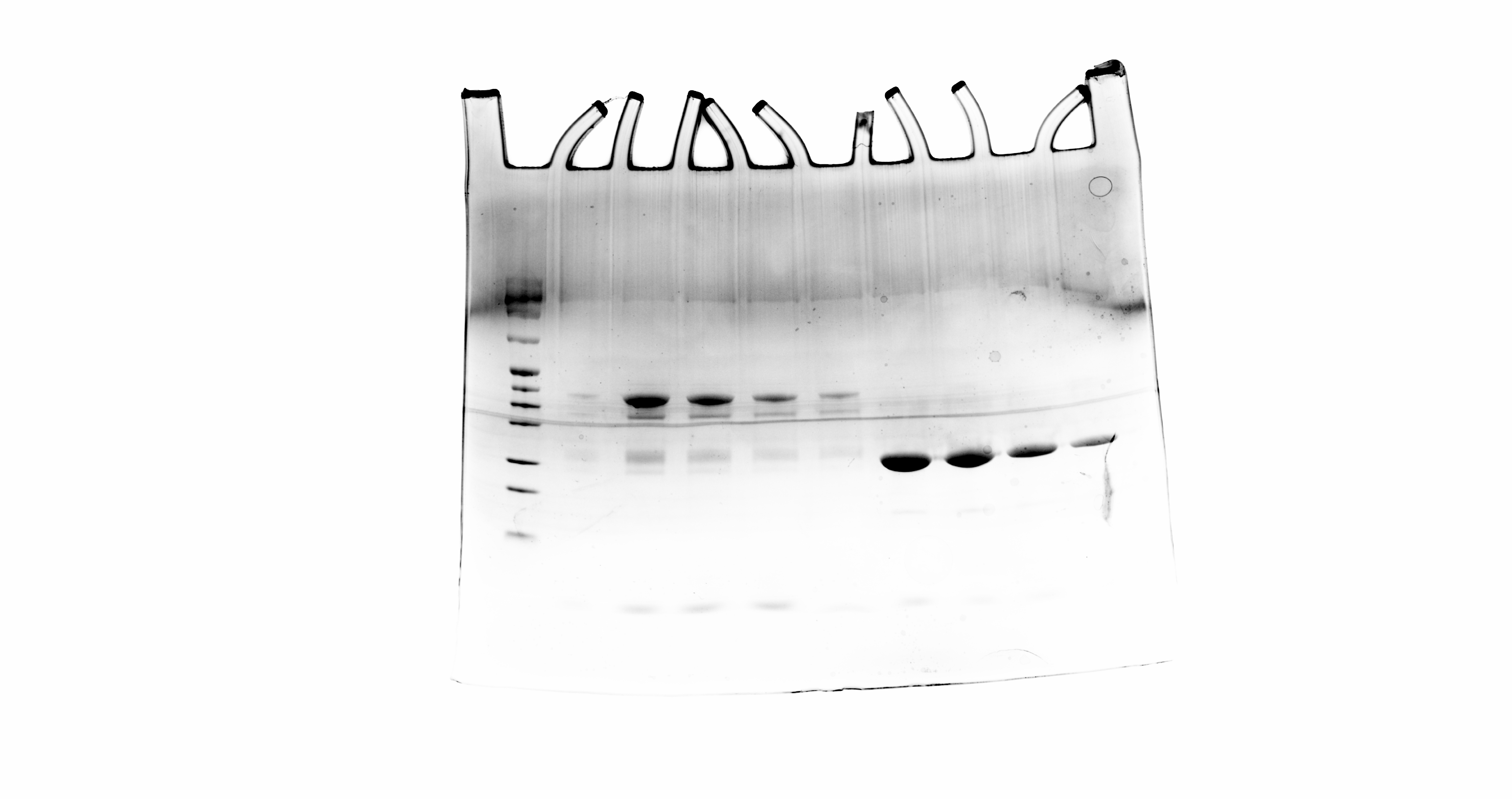

Supplement: Figure 7—figure supplement 1—source data 2. [file elife-95891-fig7-figsupp1-data2.tif]
