# Supplementary material for: Root-specific theanine metabolism and regulation at the single-cell level in tea plants (Camellia sinensis)
Source: eLife. 2024 Oct 14;13:RP95891. doi: 10.7554/eLife.95891 (PMC11473105; doi:10.7554/eLife.95891)
Supplement: Figure 7—figure supplement 1—source data 1. [file elife-95891-fig7-figsupp1-data1.pdf]

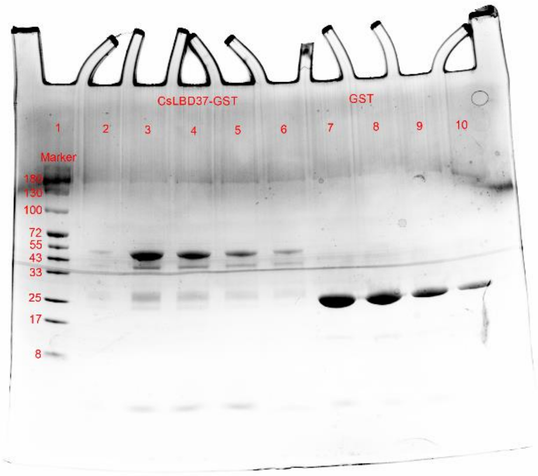

Figure 7-figure supplement 1, Source Data 1. Original membranes corresponding to Figure 7-figure supplement 1. Lanes 1 to 10, (1) protein marker; (2-6) CsLBD37-GST protein; (7-10) GST protein.
